# Semantic Knowledge Influences Prewired Hedonic Responses to Odors

**DOI:** 10.1371/journal.pone.0013878

**Published:** 2010-11-08

**Authors:** Johan Poncelet, Fanny Rinck, Anne Ziessel, Pauline Joussain, Marc Thévenet, Catherine Rouby, Moustafa Bensafi

**Affiliations:** Neurosciences Sensorielles, Comportement, Cognition, Université de Lyon and Centre National de la Recherche Scientifique, UMR5020, Lyon, France; Center for Genomic Regulation, Spain

## Abstract

**Background:**

Odor hedonic perception relies on decoding the physicochemical properties of odorant molecules and can be influenced in humans by semantic knowledge. The effect of semantic knowledge on such prewired hedonic processing over the life span has remained unclear.

**Methodology/Principal Findings:**

The present study measured hedonic response to odors in different age groups (children, teenagers, young adults, and seniors) and found that children and seniors, two age groups characterized by either low level of (children) or weak access to (seniors) odor semantic knowledge, processed odor hedonics more on the basis of their physicochemical properties. In contrast, in teenagers and young adults, who show better levels of semantic odor representation, the role of physicochemical properties was less marked.

**Conclusions/Significance:**

These findings demonstrate for the first time that the biological determinants that make an odor pleasant or unpleasant are more powerful at either end of the life span.

## Introduction

A fundamental question still unresolved in the field of olfaction is what makes an odorant smell good or bad. One theory is that acquired odor semantic knowledge is one of the important factors that determines odor hedonic valence [1], [2], [3], [4]. An alternative view is that the olfactory system is predisposed to discriminate environmental olfactory stimuli on the basis of their physicochemical properties, and that this peripheral coding partly determines odor hedonic perception [5], [6], [7]. While the two theories are not exclusive, whether such genetically predetermined hedonic encoding based on odorant structure remains “untouched” over the human life span, or whether its influence is masked by acquired olfactory semantic knowledge remains unknown.

An important aspect of olfaction is that the level of odor semantic knowledge increases from childhood to adulthood while access to it decreases with aging from adulthood onward [8]. We therefore hypothesized that during two phases of life, development and normal aging, when the level of olfactory semantic knowledge is low (childhood) or access becomes difficult (during aging), odor hedonic perception should be more tuned by the physicochemical properties of odorants.

To test this hypothesis we first recorded hedonic responses to a large set of odorants in 30 young adults (20–40 years old) and 30 seniors (60–75 years old). Participants were asked to sniff 20 odorants selected from the multidimensional physicochemical model proposed by Khan et al. [7] (see Methods). This model predicts the hedonic tone of a particular smell on the basis of its odorant structure. Thus, two groups of odorants differing in physicochemical properties were used: a) odorants supposed to be pleasant according to their physicochemical structure, or ‘A Priori Pleasant’ odorants (APP); and b) odorants supposed to be unpleasant according to their physicochemical structure, or ‘A Priori Unpleasant’ odorants (APU). Participants were asked to sniff APP and APU odorants and to give their hedonic response on a five-point pleasantness-rating scale (see Methods). Having thus provided their hedonic response, they rated odor intensity and familiarity as well as edibility (a dimension positively correlated with odor pleasantness) on a scale from 1 (not intense, not familiar, not edible) to 9 (very intense, very familiar, very edible).

## Results

**Figure 1:**
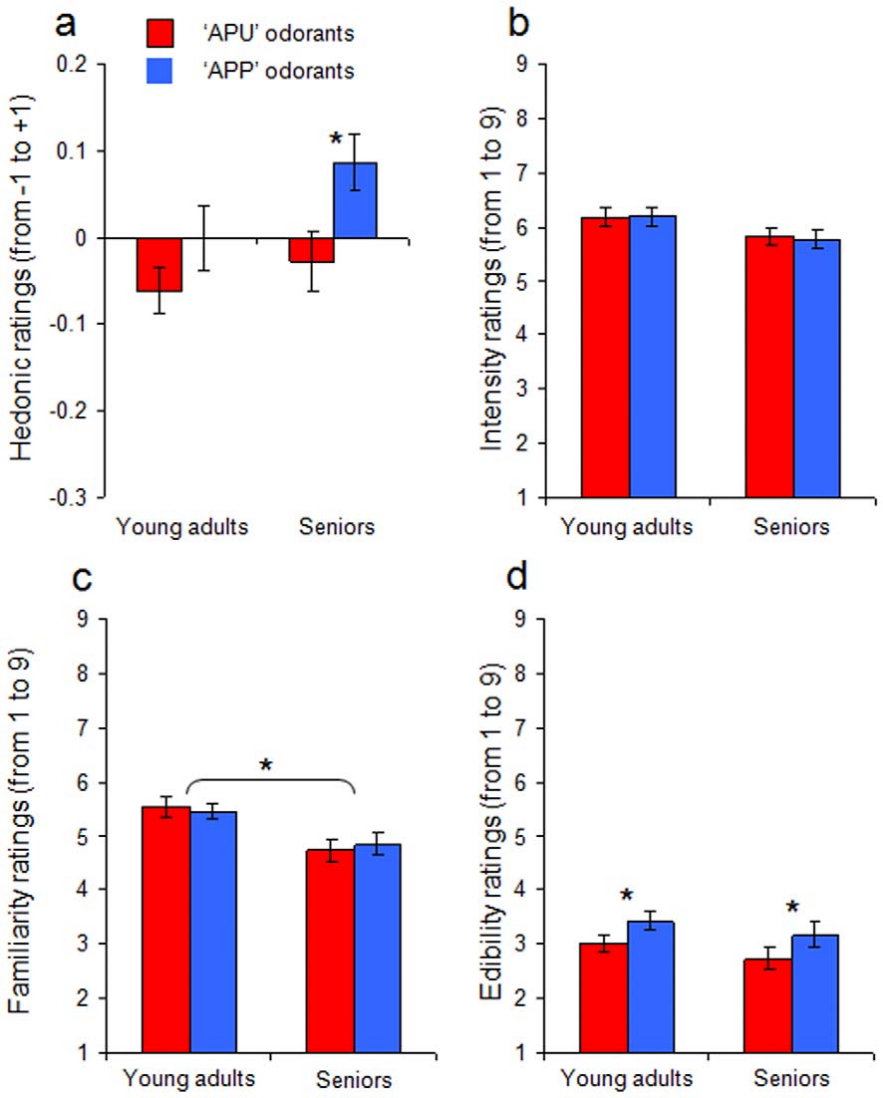
Experiment 1. a) Hedonic ratings for APU and APP odorants in young adults and seniors: Seniors (but not young adults) judged APP odorants more pleasant than APU odorants. b) Intensity ratings for APU and APP odorants in young adults and seniors: No difference in intensity ratings was observed between odorant types and between age groups. c) Familiarity ratings for APU and APP odorants in young adults and seniors: No difference in familiarity ratings was observed between odorant types; however, young adults rated odorants as more familiar than seniors. d) Edibility ratings for APU and APP odorants in young adults and seniors: APP odorants were rated as more edible than APU odorants. * significant difference at the 5% statistical significance threshold.

In line with the hypothesis of physicochemical coding of odor hedonics [5], [6], [7], when the data for the two groups of subjects were merged, APP odorants were seen to be preferred to APU odorants overall (APP: m = 0.010+/−0.017 vs. APU: m = −0.076+/−0.018; Wilcoxon test, z = 3.135; p<0.002; power = .89). However, and consistent with our predictions, analysis within each age-group revealed that APP odorants were rated as more pleasant than APU odorants specifically in seniors (Wilcoxon test, z = 2.898, p<0.05; power = 0.88) and not in young adults (Wilcoxon test, z = 1.469; p>0.05; power = 0.34) (Figure 1a). Furthermore, no significant effect of odor type (APU vs. APP) was seen for intensity (F[1,58] = .138, p>0.05; power = 0.065) (Figure 1b) or familiarity (F[1,58] = 0.005, p>0.05; power = 0.051) (Figure 1c), revealing that the effect was not due to differences in perceived intensity or familiarity. In contrast, the difference between APU and APP on edibility ratings was highly significant (F[1,58] = 16.025, p<0.0002; power = 0.987) (Figure 1d).

**Figure 2:**
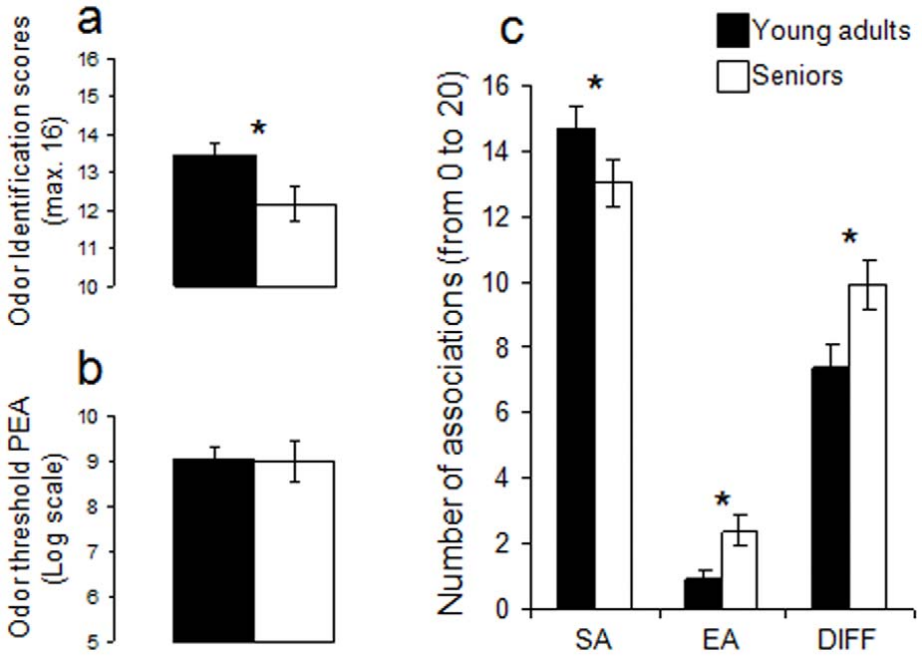
Experiment 1. a) odor identification score in young adults and seniors: seniors had lower scores of odor identification. B) odor thresholds: young adults and seniors did not differ in odor thresholds. c) odor verbalization: Young adults supplied more semantic associations (SA) and fewer emotional associations (EA) and had less difficulty in supplying any associations (DIFF) than seniors. * significant difference at the 5% statistical significance threshold.

To investigate whether the two groups differed in their semantic processing of odors, their performances were compared on a variety of olfactory tests: consistent with a difference at the semantic level, young adults exhibited better odor identification scores than seniors (F[1,58] = 5.184, p<0.03; power = 0.605) (Figure 2a) and rated all odorants as more familiar (F[1,58] = 8.349, p<0.05; power = 0.825) (Figure 1c), whereas the two groups did not differ on odor sensitivity (F[1,58] = .011, p>0.05; power = 0.051) (Figure 2b) or intensity rating (F[1,58] = 2.856, p>0.05; power = 0.366) (Figure 1b). Moreover, to further specify this difference in semantic knowledge of the APP and APU odorants used in the present study, participants were asked to verbalize on both types of stimuli by answering, after each odor trial, the question: “What does that smell make you think of?” Here, each verbalization was analyzed by dissociating “semantic associations” (e.g., “this is the smell of bananas”) from “emotional associations” (e.g., “this is very unpleasant”). Moreover, verbalizations referring to difficulty in supplying any association (e.g., “it's hard to say…”) were also analyzed (see Methods). Results revealed that young adults supplied more semantic associations than did seniors (t(58) = 1.685; p<0.05, one-tail t-test; power = 0.36) but fewer emotional associations (t(58) = 1.433; p<0.005, one-tail t-test; power = 0.71) and had less difficulty in supplying an association (t(58) = 2.489; p<0.008, one-tail t-test; power = 0.63) (Figure 2c).

**Figure 3:**
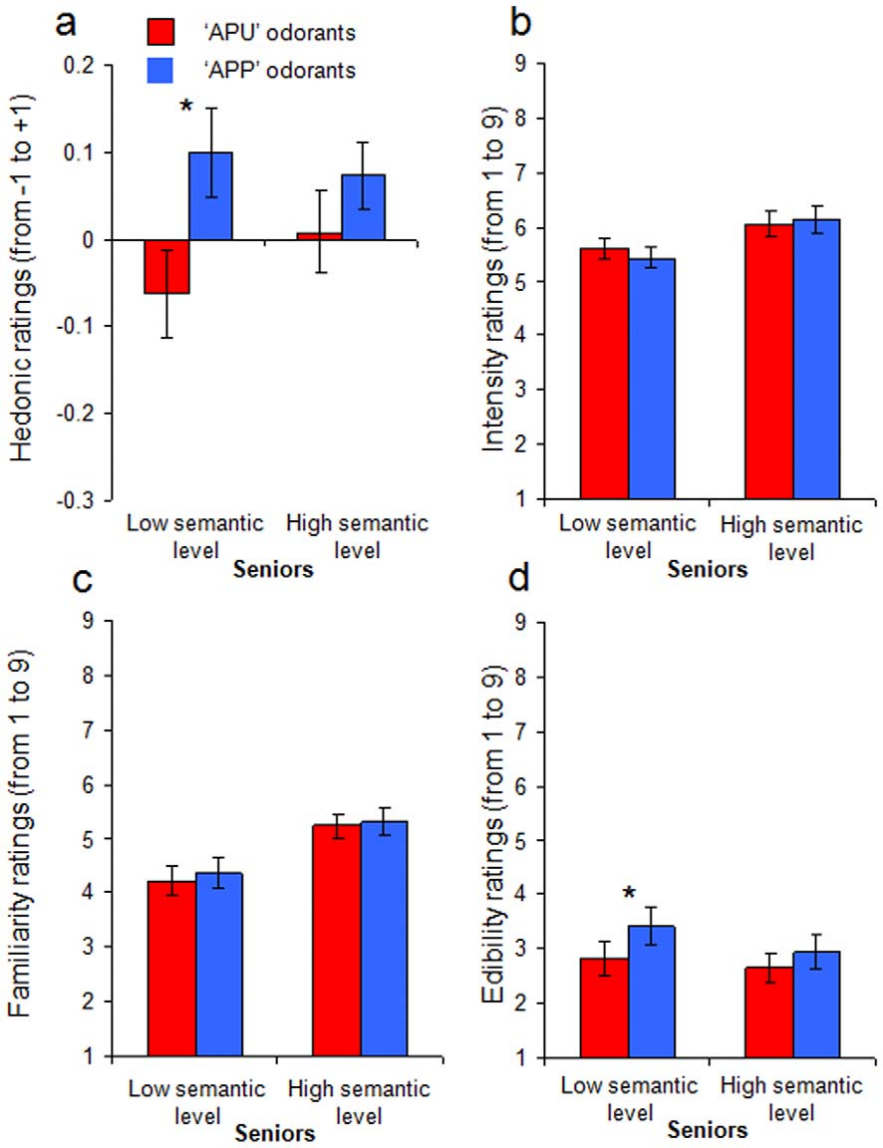
Experiment 1. Performances of seniors as function of their level of semantic knowledge. Hedonic (a), intensity (b), familiarity (c) and edibility (d) ratings for APU and APP odorants in seniors with low and high level of semantic knowledge: Seniors with a low level of semantic knowledge (but not those with high level of semantic knowledge) judged APP odorants more pleasant and edible than APU odorants. No effect of odorant type was observed for intensity and familiarity ratings. * significant difference at the 5% statistical significance threshold.

Moreover, when seniors were divided into 2 groups according to their level of odor semantic knowledge (the two sub-groups not differing in age), subjects with a lower level of semantic knowledge preferred APP odorants (vs. APU odorants) (Wilcoxon test, z = 2.726; p<0.007; power = 0.90), whereas no such difference was observed in seniors with a higher level of semantic knowledge (Wilcoxon test, z = 1.256; p>0.05; power = 0.28) (Figure 3a). Control analyses performed on other olfactory perceptual dimensions (i.e. intensity, familiarity and edibility ratings) revealed that: 1) both seniors with a low (F[1,14] = 1.613, p>0.05; power = 0.209) and high (F[1,14] = 1.000, p>0.05; power = 0.148) level of semantic knowledge estimated APP and APU odorants as equally intense (Figure 3b); 2) both seniors with a low (F[1,14] = 0.753, p>0.05; power = 0.123) and high (F[1,14] = 0.256, p>0.05; power = 0.075) level of semantic knowledge estimated APP and APU odorants as equally familiar (Figure 3c); 3) seniors with a lower level of semantic knowledge estimated APP odorants (vs. APU odorants) as more edible (F[1,14] = 5.546, p<0.04; power = 0.588), whereas no such difference was observed in seniors with a higher level of semantic knowledge (F[1,14] = 4.051, p>0.05; power = 0.455) (Figure 3d). The above mentioned effect on edibility ratings is not surprising since both pleasantness and edibility judgments of odors are perceptual dimensions that are usually positively correlated [9], [10], [11]. In other words, during normal aging, when language and semantic representations of odors are weak, the role of the physicochemical properties of odorant molecules in the genesis of olfactory affects seems to be more effective.

**Figure 4:**
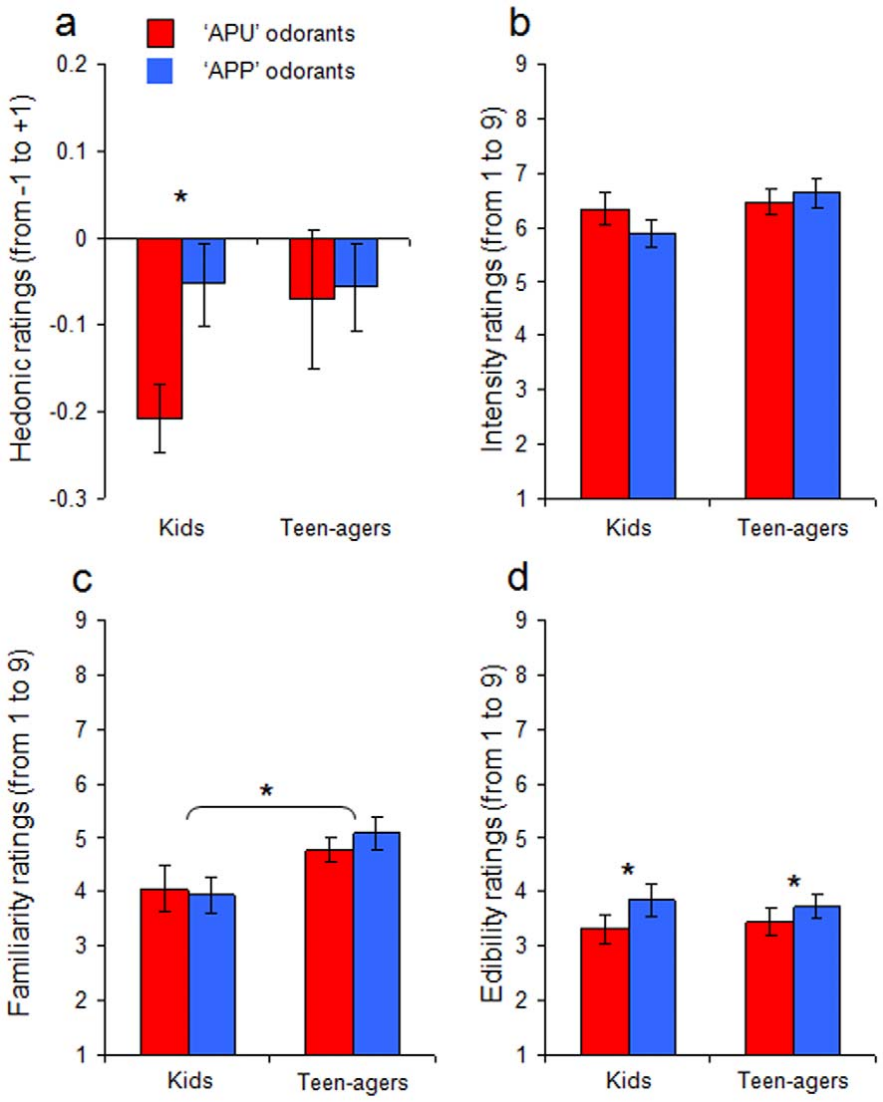
Experiment 2. a) Hedonic ratings for APU and APP odorants in kids and teen-agers: Kids (but not teen-agers) judged APP odorants more pleasant than APU odorants. b) Intensity ratings for APU and APP odorants in kids and teen-agers: No difference in intensity ratings was observed between odorant types and between age groups. c) Familiarity ratings for APU and APP odorants in kids and teen-agers: No difference in familiarity ratings was observed between odorant types; however, teen-agers rated odorants as more familiar than kids. d) Edibility ratings for APU and APP odorants in kids and teen-agers: APP odorants were rated as more edible than APU odorants. * significant difference at the 5% statistical significance threshold.

**Figure 5:**
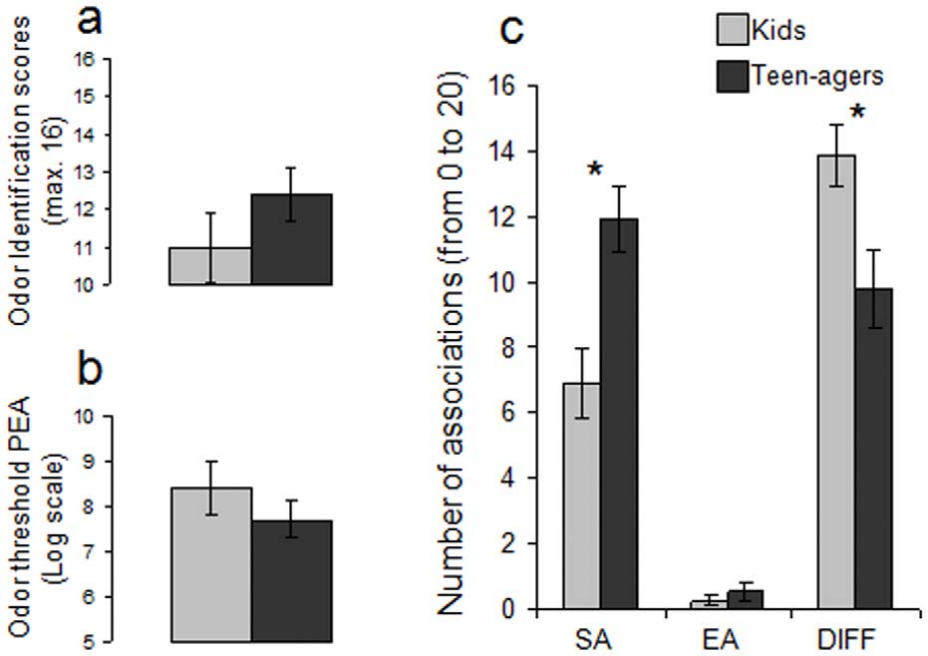
Experiment 2. Kids and teen-agers did not differ in odor identification (a) and odor threshold (b). Teen-agers supplied more semantic associations (SA) and had less difficulty in supplying any associations (DIFF) than kids (c). * significant difference at the 5% statistical significance threshold.

During childhood, olfactory identification and the level of odor semantic knowledge are relatively low as compared to adulthood [8]. Thus, if the above findings were due to semantic knowledge and not to physiological aging, one would expect that children also should have weaker olfactory semantic knowledge than teenagers, and thus discriminate odorant pleasantness more on a physicochemical basis. A second experiment tested this hypothesis by using exactly the same protocol as in the first. A group of 15 children (age range: 7–12 years) was compared to a group of 15 teenagers (age range: 13–17 years). As predicted, compared to teenagers, children supplied fewer semantic associations (t(28) = 3.459, p<0.001; power = 0.83), and judged odors less familiar (F[1,28] = 4.5, p<0.05; power = 0.525) (Figure 4c), and expressed more difficulty in supplying any semantic or emotional associations (t(28) = 2.614, p<0.008; power = 0.63) (Figure 5c). More importantly, whereas teenagers, like young adults, did not exhibit any hedonic difference between APP and APU odorants (Wilcoxon test, z = .031; p>0.05; power = 0.053), children, like seniors, rated APP odorants as more pleasant than APU odorants (Wilcoxon test, z = 2.314; p<0.03; power = 0.80) (Figure 4a). It is worth to note that children and teen-agers did differ neither in odor identification abilities (F[1,28] = 1.431, p>0.05; power = 0.200) (Figure 5a) nor in odor sensitivity (F[1,28] = 0.910, p>0.05; power = 0.145) (Figure 5b). However, as in the first experiment, the difference in hedonic ratings between APP and APU odorants in children was not explained by differences in perceived odor intensity (F[1,28] = 0.979, p>0.05; power = 0.152) (Figure 4b). In contrast, the difference between APU and APP on edibility ratings was significant (F[1,28] = 6.880, p<0.02; power = 0.721) (Figure 4d).

## Discussion

In conclusion, although some aspects of olfaction and its emotional component may be encoded early in the olfactory system [12], [13], [14] and be dependent on the physicochemical properties of odorants [5], [6], [7], olfactory perception in humans is greatly shaped by experience during childhood and adulthood [15], [16], [17], [18]. Perception of the hedonic aspect of odorants is a complex process which involves both pre-wired and learned components. Downstream of a basic encoding of odorants based on physicochemical properties, we acquire olfactory semantic knowledge. This study demonstrates for the first time that when semantic representations of objects, which are strong organizers of perception and of odor perception in particular, are relatively weak (during childhood) or their access poorer (during normal aging), the olfactory system is more tuned to the physicochemical world in interpreting the hedonic significance of odors.

Overall, these findings fit well with the lines of evidences suggesting that olfactory perception continues to be shaped by experience, learning and context during adulthood. During the human life span, odor perception and its hedonic tone are modulated by stimulus concentration [19], [20], [21], repeated [15] and previous experience [16], [17], [18], [22], current physiological status [23], stimulus exposure context (in association with trigeminal [24] or gustatory stimuli [25], [26]). Moreover, in accordance with our findings, there are several lines of evidence that olfactory semantic knowledge modulates hedonic perception of odors.

For example, jury members give higher pleasantness ratings for the odor of products presented with their brand label than for the same odors presented without [27]. Moreover, pleasantness and also intensity and familiarity judgments are enhanced when participants are able to identify the odorant source [9] or when the experimenter provides a positive name for the odorant object [28]. When verbal information about an odor is available, subjects shift their pleasantness judgment in line with the affective connotation of the label [3]. Such top-down modulation has been found even in children [29]. Moreover, Dalton [30] showed that health-related claims also influence valence: the same odorant presented as “harmful”, “healthful” or “neutral” will evoke more health symptoms when presented as dangerous. Thus labeling odors with positive or negative words (i.e., emotionally intense labels) will influence valence, emotional intensity and pleasantness ratings as compared with neutral, less emotional labels [2]. Such modulation by semantic knowledge was shown to be effective even at the neural level. Odor hedonic valence seems to be encoded at various level of the olfactory system from piriform cortex [12], [13], [14] to orbito-frontal cortex [13], [31], [32]. In an fMRI investigation, de Araujo and colleagues showed that the hedonic meaning of the label (edible-“cheese”- or not –“foot odor”) assigned to an odor differentially affected the activation pattern of one of these brain areas, namely the orbito-frontal cortex [1].

Thus, our data suggest that semantic knowledge modulates hedonic responses at both ends of the lifespan. However, one may not discard the possibility that alternative factors contribute to the above mentioned effect. For example, experience with odorant sources seems to be an important factor since familiarity ratings were lower in seniors and in children compared to adults. The observed decrease in familiarity ratings in both groups may be however sustained by different mechanisms since semantic knowledge of seniors is already constituted, whereas it is just building in children in relation with language [33], [34]. One factor that may explain the differences between age groups may be odor discrimination abilities. Indeed, discrimination deficit in odor perception is well documented in aging humans [35], [36], and could explain why seniors are impaired in accessing their semantic knowledge: matching the current perceptual input with stored representations of odors becomes problematic. In contrast, discrimination in children has been documented scarcely, but children are better at discriminating than at naming as compared to adults, and they perform like adults already at the age of 11 [37], [38]. In sum, it is possible that different processes drive the same empirical finding, and that the prevalence of physicochemical information could be due to fuzzy semantic knowledge despite of good discrimination in children, and to difficulty in matching input to a well established semantic knowledge stemming from reduced discrimination in seniors.

In conclusion, odor hedonic perception involves both pre-wired and learned components. Our phylogenetic heritage is reflected in the fact that our chemical senses – which are important for infant/parent bonding [39], search for food, and sexuality – project onto brain regions that also process basic affects and reward [40]. These affective responses to odors in humans and in other species are linked in part to the structure of odorants [5], [6], [7] and our study demonstrates for the first time that humans, thanks to their language abilities, are able to shape odor hedonics using acquired semantic representations and thus to decrease the role of the physicochemical encoding. Taken as a whole, our findings offer a new look at odor hedonic perception and its regulation by both the physicochemical properties of odorant molecules and top-down command, and open up new perspectives for understanding the mechanisms underlying modifications in olfactory perception which may affect quality of life, especially in elderly people.

## Methods

### 1. Odorant selection

Odorants were selected from the physicochemical multidimensional model proposed by Khan et al [7]. These authors applied a principal component analysis to 1,565 odorants commonly used in olfactory experiments and to 1,513 physicochemical descriptors provided by dedicated software (Dragon®). The physicochemical space generated from their analysis revealed that the principal component that explained the most variance of the original data (PC1) reflected a perceptual dimension, namely odor pleasantness.

Twenty odorants (see Table 1) were thus selected according to this physicochemical dimension (PC1): Acetophenone (ACE), Allyl Caproate (ALC), Amyl PhenylAcetate (APA), Benzyl Acetate (BENZ), Carvone-l (CARV), 1-Decanol (DEC), Dodecanal (DODEC), Diphenyl oxide (DPO), Ethyl Butyrate (ETB), Eugenol (EUG), Geraniol (GER), Guaiacol (GUA), Heptanal (HEPal), 1-Heptanol (HEPol), Hexanoic Acid (HEXoic), 3-Hexanol (HEXol), β-Ionone (ION), Isoamyl Acetate (ISO), Methyl Anthranilate (MA), and Phenyl Ethanol (PEA). All were diluted in mineral oil so as to achieve an approximate gas-phase partial pressure of 1 Pa.

**Table 1:** Odorants and their percentage (vol/vol) dilutions (1 Pa).

Acetophenone	0.56
Allyl Caproate (allyl hexanoate)	0.55
Amyl Phenyl Acetate	59.14
Benzyl Acetate	1.47
Carvone-L	2.37
1-Decanol	33.74
Dodecanal	27.74
Diphenyl Oxide	13.55
Ethyl Butyrate	0.01
Eugenol	13.12
Geraniol	21.26
Guaiacol	2.09
Heptanal	0.07
1-Heptanol	0.91
Hexanoic acid	3.63
3-Hexanol	0.08
β-Ionone	30.60
Isoamyl Acetate	0.03
MethylAnthranilate	12.65
Phenyl Ethanol	2.66

K-means clustering was applied to reduce the whole data set into two groups: odorants with a low (PC1-low) and a high PC1 value (PC1-high). According to Khan's model, PC1-low odorants should be less pleasant than PC1-high odorants. We therefore labeled them ‘A Priori Unpleasant’ (APU) and ‘A Priori Pleasant’ (APP), respectively. The results of the K-means clustering (2 clusters) were as follows: APU odorants (HEXol, MA, GUA, ACE, HEXoic, ETB, HEPal, PEA, HEPol, ISO) and APP odorants (CARV, EUG, ALC, GER, BENZ, DPO, DEC, ION, DODEC, APA).

### 2. Experimental procedure

#### 2.1. Ethics Statement

The experimental procedure was explained in great detail to the subjects, who provided written consent prior to participation. The study was conducted according to the Declaration of Helsinki and was approved by the local ethical committee (Comite de Protection des Personnes Sud-Est 2, Lyon, France).

#### 2.2. Subjects

For experiment 1, 60 participants were tested (30 young adults, 15 male and 15 female, mean age  = 29+/−5.76 yrs; and 30 seniors, 15 male and 15 female, mean age  = 67.37+/−4.24 yrs).

For experiment 2, 30 participants were tested (15 children, 8 male and 7 female, mean age  = 10.13+/−1.36 yrs; and 15 teenagers, 7 male and 8 female, mean age  = 14.33+/−1.39 yrs). Here, we compared children with teenagers (instead of young adults) in order to reduce the age difference between the groups. (An analysis comparing men and women in the two experiments on hedonic perception of odors did not reveal any significant sex difference (Mann Whitney test, Z = 1.624; p>0.05), and this factor was discarded from further analysis.).

#### 2.3. Protocol

After providing written informed consent to the procedure, which had been approved by the local ethics committee, subjects started the experiment. Testing was performed in an experimental room designed specifically for olfactory experiments. Odorants were presented in 15 ml flasks (opening diameter: 1.7 cm; height: 5.8 cm; filled with 5 ml) and were absorbed on a scentless polypropylene fabric (3×7 cm; 3M, Valley, NE, USA) to optimize evaporation and air/oil partitioning.

Once instructions had been read and the consent form signed, the experiment started. The experimenter presented the odorant flask 1 cm below the subject's nose and subjects were instructed to sniff at each presentation of a flask and rate hedonic valence using a 5-button box. After the hedonic task, participants were asked to rate odor intensity, familiarity and edibility on a scale from 1 (not at all intense, familiar, edible) to 9 (very intense, familiar, edible). Once odor ratings completed, participants were asked verbalize on each odor by answering the question “What does that smell make you think of?”

The instructions given to the subjects were as follows: “You are going to smell several odors one after the other. Your task will be to sniff each vial and then to rate odor pleasantness. For your response, here is a box with 5 buttons: the far-left button (or the far-right) means “very pleasant”, the mid-left button (or the mid-right) means “pleasant”, the middle button means “neutral”, the mid-right button (or the mid-left) means “unpleasant”, and the far-right button (or the far-left) means “very unpleasant”. Once your response given, you will estimate how intense, familiar and edible the smell was. To give your estimates, you will rate each odorant on a scale from 1 (not at all intense, familiar or edible) to 9 (very intense, familiar or edible). Then, after each of these odor ratings, you will have to explain briefly “what that smell makes you think of”.

The side of the response (i.e., “pleasant” for the left or the right button) was counterbalanced between subjects. Odorants were presented every 45 sec. In order to habituate the subject to the experimental setting, a training session consisting of a sequence of 1 to 3 empty flasks was carried out.

After the experiment, participants were asked to do two different olfactory tests:

1. Test of olfactory identification ability

Subjects' olfactory performance was estimated on the European Test of Olfactory Capabilities (ETOC) [41]. Briefly, the ETOC is based on 16 blocks of 4 flasks. Only one flask per block contains an odorant. For each block, participants are asked, first, to detect the flask containing the odor and, second, to identify the detected smell. Identification is assessed by a multiple-choice procedure in which participants have to select the correct descriptor from four proposed. The odorous solutions (volume: 5 ml) are dissolved in mineral oil and poured into a 15 ml flask (1.7 cm in diameter at the opening; 5.8 cm high). Each flask contains a synthetic absorbent (polypropylene) to optimize odor diffusion. The detection score ranges from 0 to 16 and is an indicator of sensitivity; the identification score also ranges from 0 to 16, but only odors that have been correctly detected are taken into account, thus reducing the probability of fortuitous correct identification.

2. Test of olfactory sensitivity to phenyl-ethyl-alcohol

All subjects were tested for their ability to detect smells, using a threshold test for phenyl-ethyl-alcohol (PEA, smelling like rose). In this procedure, the detection threshold is obtained by using a single-staircase procedure [42]. Here, increasing concentrations of PEA are presented. Once a given concentration has been correctly detected on five consecutive trials, a lower concentration is presented. This is the first reversal. Then, testing continues for seven reversals. The mean concentration of the last four of the seven reversals constitutes the detection threshold.

### 3. Data analysis

#### 3.1. Hedonic judgment

Comparison between hedonic ratings for APU vs. APP odorants used the Wilcoxon non-parametric test (because of the 5 choice nature of the response). To perform non-parametric tests on the data, the possible choices in the ratings were converted into numerical data (−1 for “very unpleasant”’, −0.5 for “unpleasant”, 0 for “neutral”, 0.5 for “pleasant” and 1 for “very pleasant”).

#### 3.2. Odor rating (intensity, familiarity, edibility)

The effect of groups and odor type (APP vs. APU) was analyzed using analysis of variance (ANOVA).

#### 3.3. Lexical data

To quantify the level of olfactory semantic knowledge of each subject, the 20 verbalizations produced by each subject (20 odorants were used) were analyzed on exploratory and lexical analysis. Here, each verbalization was analyzed by an experienced research linguist (FR), dissociating: 1) “semantic associations” (for example, when the subject said “This is the smell of bananas”), 2) “emotional associations” (for example, when the subject said “This is very unpleasant”), 3) verbalizations referring to difficulty in supplying any association (for example, when the subject said “It's hard to say…”). This analysis resulted, for each of this type of association, in a score from 0 (the subject did not give any association of that type) to 20 (the subject gave an association of that type for all 20 odorants used in the experiment). Since we had an a priori hypothesis regarding the direction of the effect (seniors and children would exhibit fewer semantic associations than respectively teenagers and young adults), comparison between groups was performed with a one-tail Student t-test.

#### 3.4. Splitting seniors into two groups with respectively low and high levels of semantic knowledge

In experiment 1, seniors were divided into 2 sub-groups according to their score for odor “semantic associations” (from 0 to 20: see above) using a median split procedure (given that 30 subjects were in that particular group, the median split procedure distributed the seniors into 2 groups of 15).
